# Application of Linear Gradient Solvent System in Centrifugal Partition Chromatography Facilitating Bioassay-Guided Fractionation of Yongdamsagan-Tang, Traditional Oriental Decoction

**DOI:** 10.1155/2021/7552169

**Published:** 2021-10-31

**Authors:** Ji Hoon Kim, Eun Ju Jung, Yun Jung Lee, Chul Young Kim, Je-Seung Jeon

**Affiliations:** ^1^College of Pharmacy and Institute of Pharmaceutical Science and Technology, Hanyang University, Ansan-si, Gyeonggi-do 15588, Republic of Korea; ^2^Molecular Phytobacteriology Laboratory, Infectious Disease Research Center, KRIBB, Daejeon 34141, Republic of Korea

## Abstract

As important pharmaceutical resources, traditional herbal medicines retain continuous attention. To do that, isolation and identification of bioactive molecules from traditional herbal decoction are important. However, conventional fractionation through octadecyl silica column faces irreversible sample adsorption that causes a bias in bioactivity assessment. However, liquid-liquid chromatographic system suffers tedious *K* value calculation as well as insufficient capacity in separation power when crude extract composed of widely ranging polarities. Here, we developed a comprehensive linear gradient solvent system for centrifugal partition chromatography (CPC) to aid bioassay-guided isolation. The lower aqueous phase of the *n*-hexane-acetonitrile-water (10:2:8, v/v) was used as the stationary, whereas its upper organic phase followed by the upper phase of ethyl acetate-acetonitrile-water and water-saturated *n*-butanol-acetonitrile-water in the same ratio were eluted in a linear gradient mode, thereby increasing polarity in the mobile phase. The HPLC profiling of CPC fraction showed that proposed gradient CPC was suitable to separate metabolites from Yongdamsagan-Tang, a traditional medicinal decoction made of ten herbal plants. Exhibiting a high recovery yield of 98.3%, antioxidant response element (ARE) luciferase-inducing assay in HepG2 cells indicated that the fractions composed of baicalein and wogonin, the marker natural products of *Scutellaria baicalensis,* were to be the most effective molecules from Yongdamsagan-Tang. The presented results demonstrated that bioassay-guided separation that assisted with a linear gradient CPC is an incomparable alternative to HPLC and biphasic CPC in terms of higher yield rate and redundant *K* value calculation, respectively, which led to an unbiased/time-saving separation and identification of bioactive molecules from the complex crude extract of natural products.

## 1. Introduction

Each plant species has evolved to possess an indigenous metabolite profile. Among others, plants exhibiting pharmaceutical activities have been traditionally used as folk medicine to control various ailments. In general, a mixed herbal formulation that is composed of more than two medicinal plant species was prescribed for centuries in many Asian countries in order to elicit polyvalent pharmaceutical action or to attenuate its side-effects caused by toxic metabolites by reducing the toxicity per unit weight [1–4]. For decades, pharmacognosists have focused on the isolation and identification of the metabolites from mixed herbal formulation which led to discovering various phytochemicals. However, which metabolite or plant species among others contribute the most to the specific medicinal activity remains largely unknown.

Nowadays, contemporary interpretation of the ancient medicinal herbal formulation is important. This facilitates standardization of the ancient formulation by identifying the active ingredients and its composition in a mixed decoction. Thereby, pharmaceuticals can produce a quality product of natural medicine, simply by mixing active compounds that show the same medicinal property as its origins. To date, chemical fractionation conducted by resin packed column chromatography followed by bioassay screening has long been the gold standard in natural product research as a tool to simplify the complexity of the mixture and eventually to isolate the compound(s) responsible for the target bioactivities [5]. Despite its universality, innate physicochemical properties of its solid-like packing materials such as silica and octadecyl silica (ODS C18 column) continuously raised irreversible sample binding to the column that potentially lead to lose of activity or failure in (adequate) isolation of target compound(s) from crude extract [5, 6]. Moreover, conventional bioassay-guided fractionation strategy is time- and resource-intensive.

Centrifugal partition chromatography (CPC) can be an excellent alternative to the silica-basis column chromatography. CPC is a type of support-free liquid-liquid chromatography technique that uses an immiscible biphasic solvent system to compose stationary and mobile phase. Here, the different affinity of the target compounds for each phase (partition coefficient, *K* value) plays a key role to fractionation. This feature allows a large sample loading capacity, no irreversible sample adsorption, and low risk in sample denaturation, thereby enhancing total sample recovery [7–11]. Our most recent study proposed a linear gradient CPC system to successfully fractionate ARE-inducing metabolites from *Centipeda minima* [12], a traditional medicine. In such gradient CPC, the *K* values of peaks are continuously shifting due to changing polarity of mobile phase, thereby permitting separation of compounds mixture composed of widely ranging polarities, being freed from restriction of 0.5 < *K* < 2.0 rule [13, 14] while performing similar to that of HPLC. More specifically, time consuming/tedious *K* value calculation was not needed [12, 15]. Here, we applied this CPC solvent system to separate complex natural product mixture such as traditional Oriental decoction.

Yongdamsagan-Tang (also known as Longdanxigan-Tang in China and Ryutanshakan-To in Japan) is a type of mixed decoction that consists of ten herbal medicines in a certain ratio (Table 1) and is widely used for treating hepatic disease, gall bladder, congested eyes, swelling, and pain in the ear in Korea, China, and Japan [26, 27] as an Oriental medicine prescription. Previously, various plant secondary metabolites belong to widely ranging polarities such as flavonoids, phenylethanoid glycosides, iridoid glycosides, secoiridoids glycosides, coumarins, triterpenes, and triterpenoidal saponins [28, 29] isolated and reported from Yongdamsagan-Tang. A more recent study reported that this decoction increased antioxidant enzyme activities in prostate tissues [27]. Therefore, Yongdamsagan-Tang was chosen as a model sample to carry out comprehensive linear-gradient CPC for the identification of the antioxidant response element (ARE)-inducing compounds, followed by to identify the most contributing herbal ingredients.

In the present study, together with our recent study [12], we reported a linear gradient solvent system developed for CPC fractionation and screened its active metabolites from a complex natural compound mixture such as Yongdamsagan-Tang as a model extract. Referring to the conventional liquid-liquid extraction, *n*-hexane, ethyl acetate, and water-saturated *n*-butanol in different ratios were chosen as less polar solvents in ternary biphasic solvent systems while acetonitrile was used as the polar modifier considering the settling time and upper/lower volume ratios of the two-phase solvent system. The developed gradient solvent system demonstrated unbiased activity-guided purification with an ARE-luciferase reporter assay in HepG2 cells.

## 2. Materials and Methods

### 2.1. Apparatus

An SCPC-100 + 1000 (Armen Instrument, France) apparatus and a Spot Prep II HPLC instrument (an automated HPLC system: injector, pump, UV/Vis detector, fraction collector, and digital screen, Armen Instrument, France) were combined to form a CPC system. In this study, 1000 mL rotor was applied. HPLC analyses were carried out using an Agilent 1260 HPLC system (Agilent Technologies, Waldbronn, Germany) controlled by ChemStation software. It was equipped with G1322A 1260 degasser, G1312C 1260 binary pump, G1329B autosampler, G1316A column oven, and G1315D DAD detector. NMR spectra were obtained on a Bruker model digital AVANCE III 400 spectrometer (Bruker, Germany).

### 2.2. Reagents and Materials

All solvents used for the CPC were of analytical grade and purchased from Daejung Chemical (Gyeonggi-do, Korea). The HPLC grade solvents were obtained from Fisher Scientific (Pittsburgh, PA, USA). Ultrapure water was prepared using the Milli-Q SP water purifying system. The herbal medicines used in the experiment were purchased at Kyungdong Oriental Herbal Market (Seoul, Republic of Korea), and voucher specimens were deposited in the Herbarium of the College of Pharmacy, Hanyang University.

### 2.3. Yongdamsagan-Tang Extract Preparation

To prepare Yongdamsagan-Tang, a mixed decoction composed of ten herbal medicines, each herbal medicine was grinded and mixed as described in Table 1. A total of 330 g of Yongdamsagan-Tang powder was extracted with 1000 mL of ethanol for 2 h at 80°C under reflux. Then, filtered extract was dried under a rotary evaporator and freeze dryer. The dried extract was kept at −20°C before use.

### 2.4. Ternary Biphasic Solvent System Preparation and Its Physical Properties

To find a gradient elution solvent system capable of separating compounds with a wide polarity range, various solvents in different ratios were tested. In detail, common solvents used for liquid-liquid extraction (*n*-hexane, ethyl acetate, water-saturated *n*-butanol, and water) were fixed and several polarity modifier solvents (methanol, ethanol, acetonitrile, and isopropanol) were added. The settling time, which is highly responsible for the retention of the stationary phase, was expressed as the time to form a clear bilayer between the two phases (1 : 1, v/v) when mixed. The phase ratio was calculated as the ratio of each phase after mixing the upper and the lower phase. The various ternary biphasic solvent systems and their physical properties are provided in Table S2.

### 2.5. HPLC Analysis

Yongdamsagan-Tang extract and its CPC peak fractions were analyzed by an Agilent 1260 HPLC system with an INNO C18 column (4.6 × 250 mm, 5 *μ*m, Young Jin Biochrom, Korea). The mobile phase consisted of acetonitrile (0.1% formic acid, solvent A) and water (0.1% formic acid, solvent B) in a gradient mode: 0–50 min, 10–100% A; 60 min, 100% A. The flow rate was 1 mL/min, and the injection volume was 10 *μ*L. The diode array detector (DAD) measured the UV spectrum over a range of 210 to 600 nm whilst the chromatogram of the effluents was recorded at 254 nm.

### 2.6. CPC Procedure

**Figure 1:**
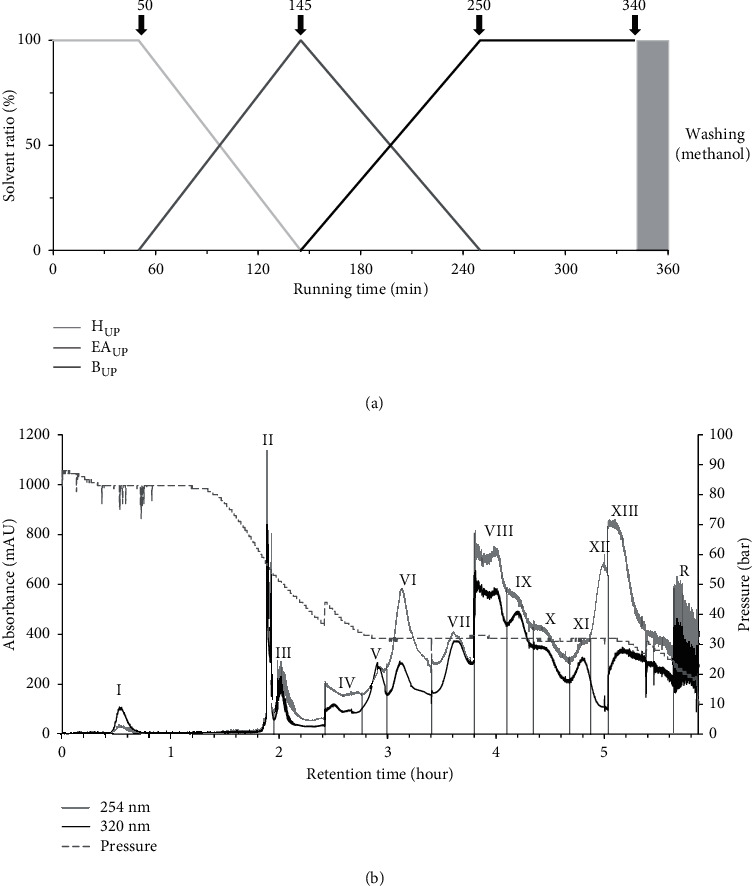
Gradient CPC for fractionation of Yongdamsagan-Tang (1.25 g). (a) Gradient mobile phase elution condition; (b) chromatogram of CPC separation at 254 nm and 320 nm. Stationary phase: lower aqueous phase of *n*-hexane-acetonitrile-water (10 : 2:8, v/v/v); flow rate: 10 mL/min; rotation speed: 900 rpm; mode ascending.

Preparation of the biphasic solvent system for the linear gradient elution mode was designed as described in [12]. To begin, the upper and lower phase solvents were prepared when the solvents reached a hydrostatic equilibration after mixing. The 1000 mL volume of the CPC rotor was filled with lower aqueous phase of *n-*hexane-acetonitrile-water (10 : 2:8, v/v/v) as the stationary phase at a flow rate of 50 mL/min in ascending mode at a rotor speed of 500 rpm. Then, the rotation speed of the rotor was accelerated to 900 rpm, and the upper organic phase as the mobile phase was carried into the rotor in descending mode at 10 mL/min. When the CPC rotor reached hydrostatic equilibrium, indicated by the outlet exuding a clear mobile phase (120 mL of stationary phase out of 1000 mL rotor volume, 88  bar), the sample solution (1.25 g of YDS extract dissolved in 5 mL of mixed upper and lower phase) was subjected to the Armen CPC system. The CPC fractionation was carried out in gradient mode with mixed mobile phase of A (upper phase of *n-*hexane-acetonitrile-water, 10 : 2:8, v/v/v), B (upper phase of ethyl acetate-acetonitrile-water, 10 : 2:8, v/v/v), and C (upper phase of water-saturated *n-*butanol-acetonitrile-water, 10 : 2:8, v/v/v) with a flow rate of 10 mL/min: 0–50 min (100% A), 50–145 min (100% A–100% B), 145–250 min (100% B–100% C), and 250–340 min (100% C). Thereafter, methanol for washing was pumped at 50 mL/min to recover all the remaining samples. The linear gradient mode is depicted in Figure 1(a).

### 2.7. Cell Culture and Viability Assay

The construction of the HepG2-ARE cells (transfected Pgl4.37 [luc2P/ARE/Hygro] (Promega)) was carried out as previous description [12, 30]. The HepG2-ARE cells were cultured in DMEM high glucose media (Hyclone, Logan, UT) supplemented with 10% FBS (Hyclone, Logan, UT), 1% penicillin-streptomycin (Hyclone, Logan, UT, USA) and 1% hygromycin B (Invitrogen, Carlsbad, CA, USA). The cell viability was examined using the 3-(4,5-dimethylthiazol-2-yl)-2,5-diphenyl tetrazolium bromide (MTT) assay. HepG2 cells stably transfected with pGL4.37 (HepG2-ARE cell) were seeded at a density of 1 × 10^5^ cells/well in 24-well plates for 24 h. After serum starvation for 12 h when they grew to approximately 80% confluency, the cells were treated with the major compounds and incubated for 24 h. Thereafter, the cells were treated with 50 *μ*L of MTT for 1 h. The formazan precipitate was dissolved in 1 mL of dimethyl-sulfoxide (DMSO), and the absorbance was measured at 570 nm using a microplate reader.

### 2.8. ARE-Inducing Activities Assay

HepG2-ARE cells were seeded at a density of 1 × 10^5^ cells/well in 24-well plates for 24 h. The cells were starved for 12 h when they reached to approximately 80% confluency and exposed the cells to crude extract, CPC fractions, and purified compounds for an additional 24 h. Then, the cells were lysed with 120 *μ*L of passive lysis buffer (Promega, Madison, WI, USA) in an ice rack and transferred in 1.5 mL tubes. The tubes were then centrifuged at 1000 rpm for 3 min. Each supernatant (30 *μ*L) in the centrifuged tube was reacted with 60 *μ*L of luciferase assay substrate (Promega, Madison, WI, USA) in a white 96-well plate. Finally, luminescence was measured by an EnSpire multimode plate reader (PerkinElmer, Waltham, MA, USA). DMSO (below 0.1%) was used as a vehicle, which was the negative control. Sulforaphane (5 *μ*M) (Calbiochem, Darmstadt, Germany) was used as the positive control.

### 2.9. Protein Assay

The protein was determined by the Pierce Micro BCA Protein Assay Kit with BSA as a standard (Pierce No. 23227; Thermo Fisher Scientific, Illinois, USA). Each standard and unknown sample lysate (10 *μ*L) was replicated into a 96-well microplate. The working reagent (200 *μ*L) was added to each well. BCA reagents A and B were mixed in a 49-to-1 volume ratio. After 30 min of incubation at 37°C, the plate was cooled at room temperature and the absorbance was measured at 562 nm on a microplate reader. The calculated value of the protein assay was used as a factor to normalize the ARE-inducing activity.

### 2.10. Statistical Analysis

All data are reported as means ± S.E. and the statistical significance of the differences between the treatments was assessed using the student's *t*-test. ^*∗*^*p* < 0.05,  ^*∗∗*^*p* < 0.01, and  ^*∗∗∗*^*p* < 0.005 were considered statistically significant. The relative luciferase activity of aglycones (baicalein and wogonin) and their glycosides (baicalin and wogonoside) to 5 *μ*M sulforaphane (%) in different combinations were analyzed with R Studio software (Version 4.1.1). To meet normality and homogeneity of variance, the data were transformed using Box-Cox using a package MASS. Differences were tested by two-way analysis of variance (ANOVA). A Tukey-HSD test was used to separate group mean values when the ANOVA was significant at *p* < 0.05 (triplicates).

## 3. Results and Discussion

### 3.1. Gradient Biphasic Solvent Systems Selection

In CPC separation, selection of an appropriate biphasic solvent system is key to success. As traditional Oriental herbal decoction such as Yongdamsagan-Tang contains a myriad of phytochemicals covering diverse polarities, solvent system satisfying isolation of compounds with polymorphous polarities is needed. To do that, a serious of ternary biphasic solvent system was tested to develop a comprehensive linear gradient solvent system for CPC. Considering the water is common for all the solvent systems, the aqueous phase was used as a stationary phase while the organic phase was used for mobile phase with increased polarities. The magnitude of polarity of organic phase was gradually enhanced with *n*-hexane, ethyl acetate, and *n*-butanol. As polarity modifier, methanol, ethanol, acetonitrile, and isopropanol were tested. The results showed that among others, acetonitrile exhibited to be a best polarity modifier in terms of the least settling time compared to others, as described in Table S2. On the other hand, isopropanol as a polar modifier was not suitable for CPC experiment due to high initial pressure, deterring mobile phase outflow, altogether gradient ternary biphasic solvent systems composed of *n*-hexane-acetonitrile-water (10 : 2:8, v/v/v), ethyl acetate-acetonitrile-water (10 : 2:8, v/v/v), and water-saturated *n*-butanol-acetonitrile-water (10 : 2:8) were chosen as apolar, semipolar, and polar conditions, respectively.

### 3.2. Separation of the CPC Gradient Elution and ARE-Inducing Activity

**Figure 2:**
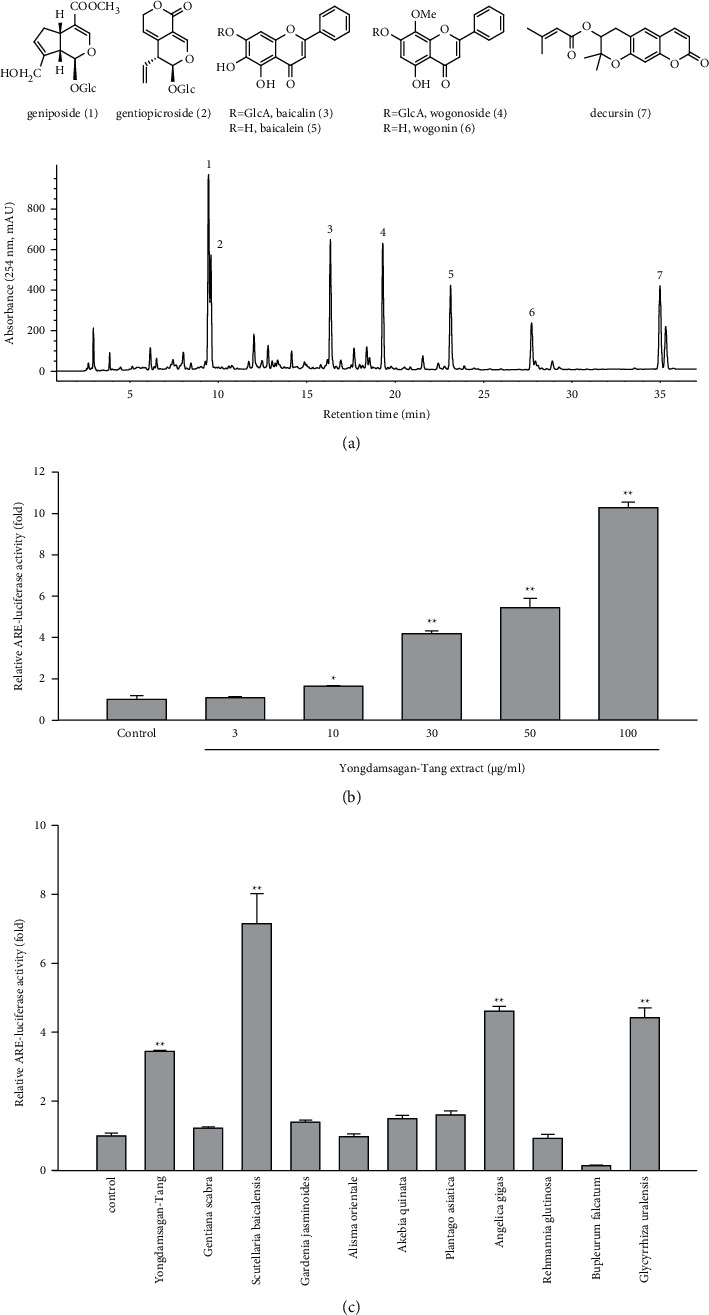
HPLC profile and relative ARE-luciferase activity of Yongdamsagan-Tang. (a) HPLC chromatogram and chemical structures of marker compounds (1–7) of Yongdamsagan-Tang; (b) relative ARE-luciferase activity of Yongdamsagan-Tang; (c) relative ARE-luciferase activity of the ingredient herbal medicines of Yongdamsagan-Tang. Data are presented as means ± S.E. (*n* = 3).  ^*∗∗*^*p* < 0.01 (compared with the vehicle-treated control).

In order to unearth ARE-inducing compounds from Yongdamsagan-Tang, we preliminary evaluated ARE-luciferase activity of each herbal medicine. Among others, *Scutellaria baicalensis*, *Angelica gigas,* and *Glycyrrhiza uralensis* exhibited to induce ARE activities (Figure 2(c)). It was shown that *S. baicalensis*, *A. gigas,* and *G. uralensis* induced an ARE via nuclear factor erythroid 2-related factor 2 (Nrf2) [31]. Here, baicalein, baicalin, and oroxylin A in *S. baicalensis* were reported to be the inducers of the Nrf2/ARE pathway, while in *A. gigas*, decursin and decursinol angelate exerted neuroprotective effects against oxidative stress which is induced by amyloid *β*-protein in PC12 cells via Nrf2-mediated upregulation of heme oxygenase-1 [32]. In *G. uralensis*, isoliquiritigenin was reported as the most potent ARE inducer [33]. However, it is difficult to reveal the active components in Yongdamsagan-Tang, since Yongdamsagan-Tang is a mixture of ten herbal medicines, which are mixtures of thousands of phytochemicals with a wide range of polarities. Therefore, we applied recently proposed comprehensive gradient CPC method [12] to search ARE-inducing compounds from Yongdamsagan-Tang.

CPC separation was carried out in a linear gradient elution of three ternary solvent systems (Figure 1(a)). For the mobile phase, the upper organic phases from *n*-hexane-acetonitrile-water (10 : 2:8, v/v), ethyl acetate-acetonitrile-water (10 : 2:8, v/v), and water-saturated *n*-butanol-acetonitrile-water (10 : 2:8) were sequentially introduced in ascending mode. As shown in Figure 1(b), fourteen fractions (**I**–**XIII** and **R**) were obtained at high resolution for 360 min operating time. The initial operating pressure was about 90 bar, then it began to decline after 80 min until 150 min, and it was then maintained at 32 bar for approximately 180 min. According to the CPC chromatogram, fourteen fractions were collected and concentrated to obtain the fractions **I** (27.5–45 min, 45.6 mg), **II** (110–117.5 min, 90.2 mg), **III** (117.5–145 min, 54.7 mg), **IV** (145–160 min, 10.3 mg), **V** (160–180 min, 11.0 mg), **VI** (180–205 min, 120.7 mg), **VII** (205–222.5 min, 21.7 mg), **VIII** (222.5–230 min, 32.0 mg), **IX** (230–260 min, 22.1 mg), **X** (260–280 min, 18.0 mg), **XI** (280–295 min, 17.1 mg), **XII** (295–302.5 min, 34.9 mg), **XIII** (302.5–340 min, 25.3 mg), and **R** (residue in rotor, 725.5 mg). Collectively, the total sum of all the CPC fractionations was 1,129.1 mg out of 1,250 mg crude extract, exhibiting a high recovery yield of 98.3%.

**Figure 3:**
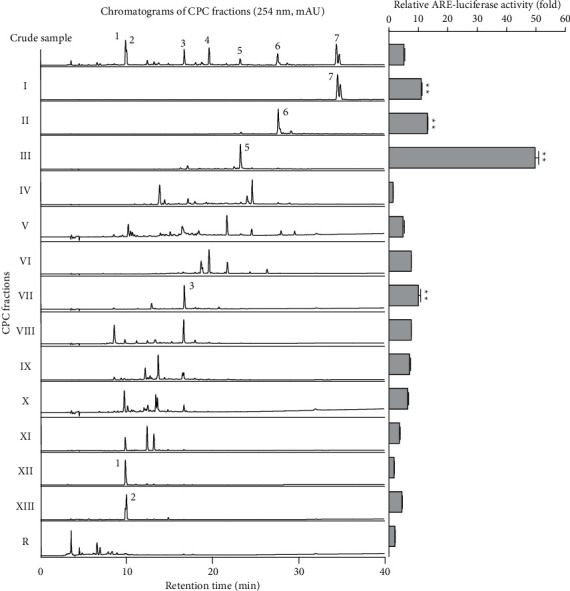
HPLC chromatograms and relative ARE-luciferase activity of CPC fractions at 30 *μ*g/mL concentrations. Each CPC fraction was analyzed by HPLC, and its ARE-inducing activity was evaluated in ARE-HepG2 cells. Data are presented as means ± S.E. (*n* = 3).  ^*∗∗*^*p* < 0.01 (compared with the vehicle-treated control).

Figure 3 displays the HPLC chromatogram of each CPC peak fraction from **I** to **R** at 254 nm. Fractions **I**, **II**, **III**, **VI**, **VII**, **XII**, and **XIII** were well corresponded to include the representative Yongdamsagan-Tang peaks **1**–**7** in reverse order. In addition, several fractions presented to be pure with a high purity such as fraction **II** (wogonin **6**, >86%); fraction **III** (baicalein **5**, 86%); fraction **VII** (baicalin **3**, 88%); and fraction **XII** (geniposide **1**, 89%). To sort out active fractions, the crude sample of Yongdamsagan-Tang and its 14 CPC fractions (**I**–**XIII** and **R** fractions) were subjected to ARE-inducing activity assay at a concentration of 30 *μ*g/mL. To begin with, fraction **III** exerted the highest activity (>50 folds increase) which is followed by fractions **II**, **I**, and **VII** (Figure 3). Interestingly, the major compounds from peak fractions **II**, **III,** and **VII** were from *S. baicalensis*, the best performing herbal medicine (in Figure 2(c)), while components from **I** were rooted in *A. gigas*, the second most effective herbal medicine to ARE-inducing activity. We then further purified the active fractions **I**–**III** and **VII** by preparative HPLC to obtain pure decursin (**7**), wogonin (**6**), baicalein (**5**), and baicalin (**3**), respectively. On the other hand, inactive but marker compounds geniposide (**1**), getiopicroside (**2**), and wogonoside (**4**) from Yongdamsagan-Tang were also purified to compare further ARE-inducing activity from the compound level. The physicochemical information of these compounds is indicated in the section Structural Information in Supplementary Materials.

**Figure 4:**
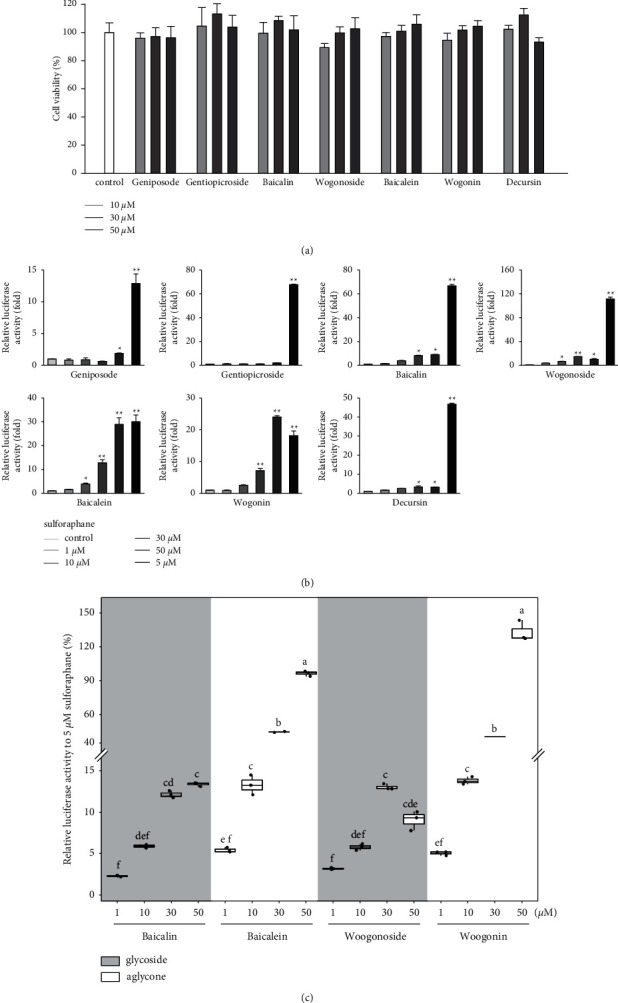
Effects of purified compounds (1–7) on ARE-luciferase activity in HepG2 cells. (a) Cytotoxicity of compounds 1–7; (b) the relative ARE-luciferase activity of isolated compounds. Data are presented as means ± S.E (*n* = 3). (c) The relative luciferase activity of baicalein and wogonin and their glycosides baicalin and wogonoside to 5 *μ*M sulforaphane (%).  ^*∗*^*p* < 0.05 and  ^*∗∗*^*p* < 0.01, (compared with the vehicle-treated control). Different letters show significant difference between the treatments (two-way ANOVA, Tukey's HSD post hoc test, *p* < 0.05, mean ± standard error, *n* = 3).

The ability of seven purified Yongdamsagan-Tang compounds to enhance ARE activity was evaluated by luciferase assay in the HepG2 cells at different concentrations. No cytotoxic activity was found (Figure 4(a)). As shown in Figures 4(b) and 4(c), baicalein (**5**) and wogonin (**6**) significantly enhanced ARE activity in a dose-dependent manner. At 50 *μ*M, baicalein (**5)** enhanced ARE activity 28.9 fold (sulforaphane 5 *μ*M, 30.0 fold), while wogonin (**6**) induced 24.0 fold (sulforaphane 5 *μ*M, 18.1 fold). In addition, baicalin (**3**) and wogonoside (**4**), glycosides of baicalein and wogonin, respectively, also exhibited induced ARE activity, but their potencies were less than those of aglycones (Figure 4(c)). Geniposide (**1**) and decursin (**7**) also presented ARE-inducing activity. Overall, the results showed that baicalein (**5**) and wogonin (**6**) from *S. baicalensis* were major contributor to ARE-inducing activity of Yongdamsagan-Tang and this was in line with ARE-luciferase activity of each medicinal plant, as shown in Figure 2(c). Other medicinal plant species *A. gigas* showed a moderate contribution to ARE activity of Yongdamsagan-Tang while *G. uralensis* was deemed to have least or no contribution to this decoction.

Our CPC-aided activity-guided fractionation clearly indicated that baicalein (**5**) and wogonin (**6**) are major components responsible for enhancing ARE activity of Yongdamsagan-Tang, a traditional decoction of ten traditional medicines. Here, together with our previous study [12], a linear gradient CPC system was extensively applied to fractionate a complex compounds pool composed of diverse polarities such as mixed herbal decoctions. Without irreversible sample adsorption or loss, this separation system provided the higher recovery rate which in turn contributed to unbiased activity-guided fractionation. Thus, our study highlights that linear gradient CPC system is an effective alternative to preparative HPLC in activity-guided fraction practice and this platform can be associated to other bioactivity assays as a reliable means of unbiased fractionation tool.

## 4. Conclusions

In this study, a comprehensive gradient solvent system for CPC was developed to aid activity-guided fractionation of Yongdamsagan-Tang, a mixture of ten herbal medicines. It was shown that a gradient elution of the upper phases of three ternary solvent system with *n*-hexane-acetonitrile-water, ethyl acetate-acetonitrile-water, and water-saturated *n*-butanol-acetonitrile-water (each 10 : 2:8, v/v/v) enabled CPC to fractionate Yongdamsagan-Tang, and among others, baicalein (**5**) and wogonin (**6**) were the most prominent ARE inducer in our assay system. Considering that CPC has no irreversible sample adsorption, this platform can be an effective method that aids unbiased screening of bioactive natural products from complex metabolite mixtures such as traditional Oriental decoction.

## Figures and Tables

**Table 1:** Composition of Yongdamsagan-Tang.

Ratin name	Scientific name	Ratio (g)	Marker compound	Reference
Gentianae Scabrae Radix et Rhizoma	*Gentiana scabra* Bunge	30	Gentiopicroside	[16]
Scutellariae Radix	*Scutellaria baicalensis* Georgi	40	Baicalin, wogonoside, baicalein, wogonin	[17]
Gardeniae Fructus	*Gardenia jasminoides* J.Ellis	30	Geniposide, gardenoside, crocetin	[18]
Alismatis Rhizoma	*Alisma plantago-aquatica* subsp. *Orientale* (Sam.) Sam.	30	Alisol A, alisol A 24-acetate, alisol B 24-acetate	[19]
Akebia Caulis	*Akebia quinata* (Houtt.) Decne.	40	23-Hydroxy-3*β*-[(*O*-*α*-L-rhamnopyranosyl (1⟶2)-*α*-L-arabinopyranosyl)oxy]olean12-en-28-oic acid *O*-*α*-L-rhamnopyranosyl-(1⟶4)-O-*β*-D-glucopyranosyl-(1⟶6)-*β*-D-glucopyranosyl ester	[20]
Plantaginis Semen	*Plantago asiatica* L.	40	Plantamajoside, acetoside	[21]
Angelicae Gigantis Radix	*Angelica gigas* Nakai	30	Decursin, decursinol angelate	[22]
Rehmanniae Radix	*Rehmannia glutinosa* (Gaertn.) DC.	50	Aucubin, Leonuride, acteoside, echinacoside	[23]
Bupleuri Radix	*Bupleurum falcatum* L.	30	Saikosaponin a	[24]
Glycyrrhizae Radix et Rhizoma	*Glycyrrhiza uralensis* Fisch.	10	Liquiritin, glycyrrhizic acid	[25]

## Data Availability

The data used to support the findings of this study are included within the article.

## Supplementary Materials

Supplementary Materials Figure S1. HPLC chromatograms of Yongdamsagan-Tang and its individual herbal components. Table S1. The partition coefficients (K values) of major compounds in Yongdamsagan-Tang. Table S2. Phase ratios and settling times of each ternary biphasic solvent system tested for a linear gradient solvent system. Physicochemical/structural information of purified metabolites from Yongdamsagan-Tang.
