# Supplementary material for: Application of Linear Gradient Solvent System in Centrifugal Partition Chromatography Facilitating Bioassay-Guided Fractionation of Yongdamsagan-Tang, Traditional Oriental Decoction
Source: Evid Based Complement Alternat Med. 2021 Oct 31;2021:7552169. doi: 10.1155/2021/7552169 (PMC8572592; doi:10.1155/2021/7552169)
Supplement: Supplementary Materials — Figure S1. HPLC chromatograms of Yongdamsagan-Tang and its individual herbal components. Table S1. The partition coefficients (K values) of major compounds in Yongdamsagan-Tang. Table S2. Phase ratios and settling times of each ternary biphasic solvent system tested for a linear gradient solvent system. Physicochemical/structural information of purified metabolites from Yongdamsagan-Tang. [file 7552169.f1.docx]

**Application of linear gradient solvent system in centrifugal partition chromatography facilitating bioassay-guided fractionation of Yongdamsagan-Tang, traditional oriental decoction**

Ji Hoon Kim^1^, Eun Ju Jung^1^, Yun Jung Lee^1^, Chul Young Kim^1*^, and Je-Seung Jeon^1,2*^

^1^College of Pharmacy and Institute of Pharmaceutical Science and Technology, Hanyang University, Ansan, Gyeonggi-do 15588, Republic of Korea
 ^2^Molecular Phytobacteriology Laboratory, Infectious Disease Research Center, KRIBB, Daejeon 34141, Republic of Korea

*Correspondence should be addressed to Chul Young Kim and Je-Seung Jeon; chulykim@hanyang.ac.kr and jsjeoncy@gmail.com

***Determination of partition coefficient***

The *K* values were calculated as a following method. Briefly, 10-15 mg of crude extracts were percolated with 1.8 mL of each phase (1:1, v/v) of two-phased immiscible solvent in a 2 mL tube. After shaking the tube, the equal volume of the upper and the lower phase were analyzed individually by HPLC to evaluate the optimal biphasic solvent. The *K* values of target compounds were calculated by following equation:
*K* value = the peak areas of mobile_upper layer_ phase / the peak areas of stationary_lower layer_ phase.

**Table S1**. The partition coefficients (*K* values) of major compounds in Yongdamsagan-Tang.

| Solvent systems | *K* values | | | | | | |
| --- | --- | --- | --- | --- | --- | --- | --- |
|  | geniposide (**1**) | gentiopicroside (**2**) | baicalin (**3**) | wogonoside (**4**) | baicalein (**5**) | wogonin (**6**) | decursin **(7**) |
| ^U^H/^1L^H | -* | - | - | - | 3.84 | 3.26 | 0.14 |
| ^U^EA/^2L^H | 13.76 | 22.99 | 2.76 | 1.07 | 0.12 | 0.15 | 0.17 |
| ^U^B/^3L^H | 1.17 | 1.15 | 0.60 | 0.47 | 0.04 | 0.03 | 0.02 |

^U^H: the upper layer of *n*-hexane-acetonitrile-water (10:2:8, v/v); ^1L^H : the lower layer of *n*-hexane-acetonitrile-water (10:2:8, v/v/v); ^U^EA: the upper layer of ethyl acetate-acetonitrile-water (10:2:8, v/v/v); ^2L^H: the lower layer of mixtures ^1L^H and ^U^EA.; ^U^B: the upper layer of water-saturated *n*-butanol-acetonitrile-water (10:2:8, v/v/v); ^3L^H: the lower layer mixtures ^2L^H and ^U^B; *, not calculated.

**Table S2**. Phase ratios and settling times of each ternary biphasic solvent system tested for a linear gradient solvent system. Our most recent publication [1] shares the following solvent system.

| Less polar solvent systems | | | | Medium polar solvent systems | | | | Polar solvent systems | | | |
| --- | --- | --- | --- | --- | --- | --- | --- | --- | --- | --- | --- |
| Solvent system | Volume ratios (v/v/v) | Phase ratios | Settling time | Solvent system | Volume ratios (v/v/v) | Phase ratios | Settling time | Solvent system | Volume ratios (v/v/v) | Phase ratios | Settling time |
| H/M/W | 10:1:9 | 50/50 | 12 s | EA/M/W | 10:1:9 | 58/42 | 19 s | B/M/W | 10:1:9 | 50/50 | 26 s |
|  | 10:2:8 | 50/50 | 12 s |  | 10:2:8 | 58/42 | 20 s |  | 10:2:8 | 56/44 | 3 m 17 s |
| H/E/W | 10:1:9 | 50/50 | > 4 m | EA/E/W | 10:1:9 | 48/52 | 13 s | B/E/W | 10:1:9 | 48/52 | 1 m 30 s |
|  | 10:2:8 | 49/51 | 50 s |  | 10:2:8 | 52/48 | 19 s |  | 10:2:8 | 57/43 | > 4 m |
| H/I/W | 10:1:9 | 50/50 | > 4 m | EA/I/W | 10:1:9 | 50/50 | 14 s | B/I/W | 10:1:9 | 50/50 | 15 s |
|  | 10:2:8 | 51/49 | 24 s |  | 10:2:8 | 54/46 | 19 s |  | 10:2:8 | 58/42 | 24 s |
|  | 10:3:7 | 52/48 | 20 s |  | 10:3:7 | 62/38 | 22 s |  | 10:3:7 | 70/30 | 37 s |
|  | 10:4:6 | 54/46 | 17 s |  | 10:4:6 | 74/26 | 27 s |  | 10:4:6 | 96/4 | 1 m 40 s |
| H/A/W | 10:1:9 | 50/50 | 14 s | EA/A/W | 10:1:9 | 50/50 | 10 s | B/A/W | 10:1:9 | 50/50 | 36 s |
|  | 10:2:8 | 50/50 | 11 s |  | 10:2:8 | 56/44 | 11 s |  | 10:2:8 | 60/40 | 43 s |

H: *n*-hexane, M: methanol, W: water E: ethanol, I: isopropanol, A: acetonitrile, EA: ethyl acetate, B: water-saturated *n*-butanol.


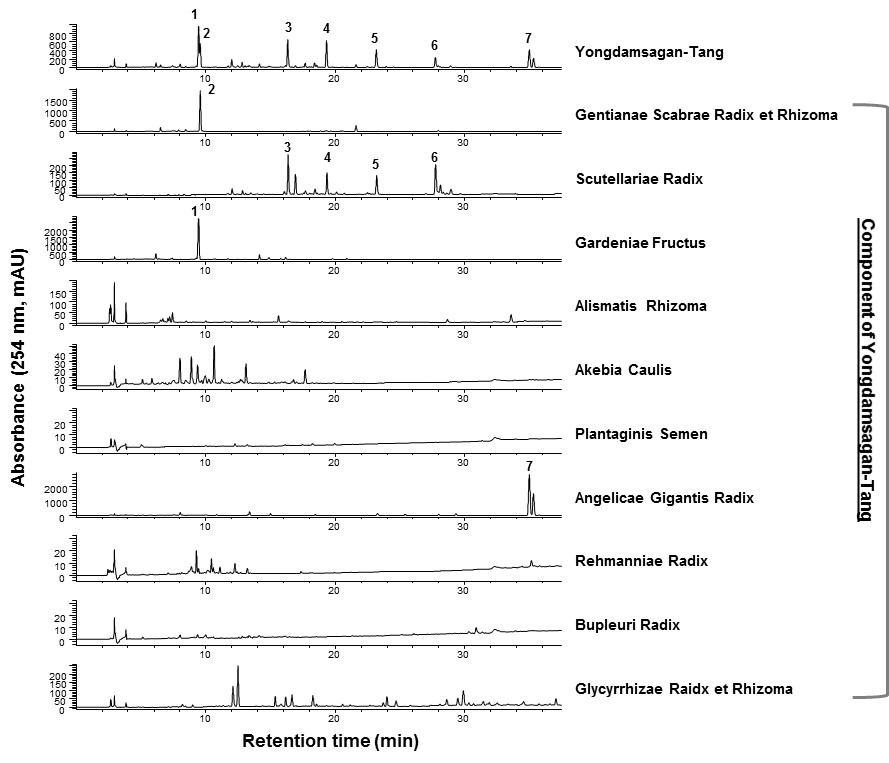


**Figure S1**. HPLC chromatograms of Yongdamsagan-Tang and its individual herbal components.

Yongdamsagan-Tang extract and individual herbal medicines were analyzed by an Agilent 1260 HPLC system with an Inno C18 column (4.6 x 250 mm, 5 μm, YoungJin Biochrom, Korea). The mobile phase consisted of acetonitrile (0.1 % formic acid, solvent A) and water (0.1 % formic acid, solvent B) in a gradient mode: 0-50 min, 10-100 % A; 60 min, 100 % A. The flow rate was 1 mL/min, whereas the injection volume was 10 μL. The chromatogram of the effluents was recorded at 254 nm.

***Structural identification of major compounds by NMR***

^1^H NMR and ^13^C NMR spectra were measured on a Bruker model digital AVANCE III 400 spectrophotometer (Bruker, Germany). The chemical shifts are reported in ppm (*δ* scale) and all coupling constants (*J*) values are in hertz (Hz). After comparison of their spectroscopic date with those reported in the literature [2-6], the purified compounds were identified as geniposide (**1**), gentiopicroside (**2**), baicalin (**3**), wogonoside (**4**), baicalein (**5**), and wogonin (**6**). Decursin (**7**) was obtained from Sigma-Aldrich (St Louis, MO) for ARE-luciferase assay.

**Geniposide (1)**: ^1^H NMR (400 MHz, DMSO-d_6_) δ 7.47 (d, *J* = 1.1 Hz, 1H, H-3), 5.68 (s, 1H, H-7), 5.10 (dd, *J* = 11.8, 6.1 Hz, H-1), 4.75 (t, *J* = 5.4 Hz, 1H, H-1'), 4.13 (dd, *J* = 15.3, 2.2 Hz, 1H, H-10b), 3.97 (dd, *J* = 15.0, 2.8 Hz, 1H, H-10a), 3.67 (dd, *J* = 6.3, 1.8 Hz, 1H, H-6'b), 3.64 (s, 3H, OCH­_3_), 3.41 (dt, *J* = 14.4, 5.7 Hz, 1H, H-3'), 3.20 – 3.00 (m, 6H, Glc 2'-6'), 2.70 (m, 1H, H-6a)2.66 (t, *J* = 7.8 Hz, 1H, H-9), 2.04 (ddd, *J* = 16.2, 4.9, 2.1 Hz, 1H, H-6b). ^13^C-NMR (100 MHz, DMSO-d_6_) δ 167.38 (C-11), 152.06 (C-3), 144.57 (C-8), 125.89 (C-7), 111.37 (C-4), 99.02 (C-1), 96.16 (C-1'), 77.74 (C-5'), 77.08 (C-3'), 73.76 (C-2'), 70.41 (C-4'), 61.42 (C-6'), 59.81 (C-10), 51.51 (OCH_3_), 46.31 (C-9), 38.43 (C-6), 34.94 (C-5).

**Gentiopicroside (2)**: ^1^H NMR (400 MHz, CD_3_OD) δ 7.45 (d, *J* = 1.3 Hz, 1H, H-3), 5.76 (ddd, *J* = 17.2, 10.3, 6.9 Hz, 1H, H-8), 5.67 (d, *J* = 2.9 Hz, 1H, H-1), 5.64 – 5.59 (m, 1H, H-6), 5.26 (t, *J* = 1.4 Hz, 1H, H-10a), 5.22 (dd, *J* = 3.2, 1.4 Hz, 1H, H-10b), 5.12 – 4.94 (m, 2H, H-7a, H-7b), 4.65 (d, *J* = 7.9 Hz, 1H, H-1'), 3.90 (dd, *J* = 11.9, 2.1 Hz, 1H, H-6'a), 3.65 (dd, *J* = 11.9, 6.2 Hz, 1H, H-6'b), 3.40 – 3.32 (m, 6H, Glc 2'-6'), 3.24 (dd, *J* = 9.5, 8.9 Hz, 1H, H-4'), 3.15 (dd, *J* = 9.1, 8.0 Hz, 1H, H-2'). ^13^C NMR (100 MHz, CD_3_OD) δ 164.93 (C-11), 149.24 (C-3), 133.62 (C-8), 125.58 (C-5), 117.12 (C-10), 115.82 (C-6), 103.54 (C-4), 98.77 (C-1'), 97.09 (C-1), 77.02 (C-3'), 76.56 (C-5'), 73.14 (C-2'), 70.12 (C-4'), 69.53 (C-7), 61.37 (C-6'), 45.21 (C-9).

**Baicalin (3)**: ^1^H NMR (400 MHz, DMSO-d_6_) δ 12.59 (s, 1H, 5-OH), 8.69 (s, 1H, 6-OH), 8.12 – 8.04 (m, 2H, H-2', H-6'), 7.60 (d, *J* = 7.4 Hz, 3H, H-3', H-4', H-5'), 7.05 (s, 1H, H-3), 7.01 (s, 1H, H-8), 5.25 (d, 1H, H-1''), 4.08 (d, 1H, H-5''), 3.45 – 3.35 (m, 3H, H-2'', H-3'', H-4''). ^13^C NMR (100 MHz, DMSO-d_6_) δ 183.03 (C-4), 170.56 (C-6''), 164.01 (C-2), 151.76 (C-7), 149.68 (C-9), 147.24 (C-5), 132.54 (C-4'), 131.32 (C-1'), 131.08 (C-6), 129.65 (C-3', C-5'), 126.86 (C-2', C-6'), 106.60 (C-10), 105.23 (C-3), 100.39 (C-1''), 94.20 (C-8), 75.95 (C-3''), 75.72 (C-5''), 73.27 (C-2''), 71.79 (C-4'').

**Wogonoside (4)**: ^1^H NMR (400 MHz, DMSO-d_6_) δ 12.57 (s, 1H, 5-OH), 8.13 – 8.08 (m, 2H, H-2', H-6'), 7.64 (d, *J* = 7.1 Hz, 3H, H-3', H-4', H-5'), 7.09 (s, 1H, H-3), 6.73 (s, 1H, H-6), 3.91 (s, 3H, OCH_3_), 5.29 (d, 1H, H-1''), 4.02 (d, 1H, H-5''), 3.60 – 3.25 (m, 3H, H-2'', H-3'', H-4''). ^13^C NMR (100 MHz, DMSO-d_6_) δ 182.87 (C-4), 170.69 (C-6''), 164.05 (C-2), 156.49 (C-7), 156.46 (C-9), 149.68 (C-5), 132.77 (C-4'), 131.18 (C-1'), 129.78 (C-8, C-5', C-3'), 126.89 (C-2', C-6'), 105.82 (C-3), 105.72 (C-10), 100.10 (C-1''), 99.13 (C-6), 76.34 (C-5''), 75.66 (C-3''), 73.37 (C-2''), 71.77 (C-4''), 61.88 (OCH_3_).

**Baicalein (5)**: ^1^H NMR (400 MHz, DMSO-d_6_) δ 12.51 (s, 1H, 5-OH), 8.08 (d, *J* = 7.9 Hz, 2H, H-2', H-6'), 7.62 (d, *J* = 6.7 Hz, 3H, H-3', H-4', H-5'), 7.01 (s, 1H, H-8), 6.32 (s, 1H, H-3). ^13^C NMR (100 MHz, DMSO-d_6_) δ 182.52 (C-4), 163.46 (C-2), 157.98 (C-7), 156.71 (C-9), 150.09 (C-5), 132.57 (C-4'), 131.32 (C-1'), 129.75 (C-6), 128.26 (C-3', C-5'), 126.75 (C-2', C-6'), 105.52 (C-10), 104.18 (C-3), 99.65 (C-8).

**Wogonin (6)**: ^1^H NMR (400 MHz, DMSO-d_6_) δ 12.51 (s, 1H, 5-OH), 8.08 (dd, *J* = 7.6, 1.8 Hz, 2H, H-2', H-6'), 7.64 – 7.60 (m, 3H, H-3', H-4', H-5'), 7.00 (s, 1H, H-6), 6.30 (s, 1H, H-3), 3.85 (s, 3H, OCH_3_). ^13^C NMR (100 MHz, DMSO-d_6_) δ 182.50 (C-4), 163.43 (C-2), 158.15 (C-7), 156.72 (C-9), 150.09 (C-5), 132.57 (C-4'), 131.34 (C-1'), 129.75 (C-3', C-5'), 128.29 (C-8), 126.76 (C-2', C-6'), 105.52 (C-3), 104.10 (C-10), 99.69 (C-6), 61.50 (OCH_3_).

**References**

1. Kim, J. H.; Jung, E. J.; Lee, Y. J.; Gao, E. M.; Syed, A. S.; Kim, C. Y., Bioassay-Guided Separation of *Centipeda minima* Using Comprehensive Linear Gradient Centrifugal Partition Chromatography. *Molecules* **2020,** 25, (13), 3077.

2. Inouye, H.; Takeda, Y.; Nishimura, H., Two new iridoid glucosides from *Gardenia jasminoides* fruits. *Phytochemistry* **1974,** 13, (10), 2219-2224.

3. Isobe, T.; Ohsaki, A.; Nagata, K., Antibacterial constituents against Helicobacter pylori of Brazilian medicinal plant, Pariparoba. *Yakugaku zasshi: Journal of the Pharmaceutical Society of Japan* **2002,** 122, (4), 291-294.

4. Wu, S.; Sun, A.; Liu, R., Separation and purification of baicalin and wogonoside from the Chinese medicinal plant *Scutellaria baicalensis* Georgi by high-speed counter-current chromatography. *Journal of Chromatography A* **2005,** 1066, (1-2), 243-247.

5. HAJI, M. H.; DIJOUX, F. M.; Mariotte, A.; Amanzadeh, Y.; SADAT, E. S.; GHAZI, K. M.; MOZAFARIAN, V., Phytochemical study of *Swertia longifolia*. **2008**.

6. Yuan, Y.; Hou, W.; Tang, M.; Luo, H.; Chen, L.-J.; Guan, Y. H.; Sutherland, I. A., Separation of flavonoids from the leaves of *Oroxylum indicum* by HSCCC. *Chromatographia* **2008,** 68, (11-12), 885-892.
